# Analysis on Coupled Vibration of a Radially Polarized Piezoelectric Cylindrical Transducer

**DOI:** 10.3390/s17122850

**Published:** 2017-12-08

**Authors:** Jie Xu, Shuyu Lin, Yan Ma, Yifan Tang

**Affiliations:** Shaanxi Key Laboratory of Ultrasonics, Institute of Applied Acoustics, Shaanxi Normal University, Xi’an 710119, China; jiexu2013@snnu.edu.cn (J.X.); mayan1982@snnu.edu.cn (Y.M.); dg1722059@smail.nju.edu.cn (Y.T.)

**Keywords:** piezoelectric cylindrical transducer, coupled vibration, mechanical coupling coefficient, resonance frequency

## Abstract

Coupled vibration of a radially polarized piezoelectric cylindrical transducer is analyzed with the mechanical coupling coefficient method. The method has been utilized to analyze the metal cylindrical transducer and the axially polarized piezoelectric cylindrical transducer. In this method, the mechanical coupling coefficient is introduced and defined as the stress ratio in different directions. Coupled vibration of the cylindrical transducer is regarded as the interaction of the plane radial vibration of a ring and the longitudinal vibration of a tube. For the radially polarized piezoelectric cylindrical transducer, the radial and longitudinal electric admittances as functions of mechanical coupling coefficients and angular frequencies are derived, respectively. The resonance frequency equations are obtained. The dependence of resonance frequency and mechanical coupling coefficient on aspect ratio is studied. Vibrational distributions on the surfaces of the cylindrical transducer are presented with experimental measurement. On the support of experiments, this work is verified and provides a theoretical foundation for the analysis and design of the radially polarized piezoelectric cylindrical transducer.

## 1. Introduction

Radially polarized piezoelectric cylindrical transducers are widely used for filters, sensors, transmitters, and actuators, as well as in the field of nondestructive testing, medical diagnostics, underwater signaling, and ultrasonic motors [1,2,3,4,5]. For the theoretical analyses of the piezoelectric cylindrical transducer, Shuaijun Wang studied the ring-type and tube-type piezoelectric transducers polarized in the radial direction [6,7]. The vibration of the piezoelectric ring is along the plane radial direction, and the vibration of the piezoelectric tube is along the longitudinal direction. To increase the radiation area, augment the radiation range and realize the radial and longitudinal propagation at the same time, the large dimension and finite length piezoelectric cylindrical transducer is studied. Ebenezer described the piezoelectric thin shell theoretical model to analyze the radially polarized ceramic cylinder [8,9]. Larbi developed accurate piezoelectric shell finite elements of radially polarized multilayer piezoelectric hollow cylinder [10]. In these reports, the shell theory [11,12,13,14] and the membrane theory [15] are used, which neglect the radial stress and strain in piezoelectric equations [16]. It is more preferable for the analysis of the thin-wall transducer. When the thickness of the wall of the piezoelectric transducer is without limitation, the shell and the membrane theory are not recommended. In this case, the vibration of the piezoelectric cylindrical transducer is a complex coupled vibration, and the radial, circumferential, and axial stresses and strains are all considered.

To analyze the complex coupled vibration of the transducer, Giebe and Blechshmidt firstly proposed that vibration of the cylindrical transducer was considered as a dynamical coupling between two partial mechanical systems [17]. This thought provided an effective way to study the coupled vibration of large dimension and finite length cylindrical transducers. According to this thought, Boris Aronov used the energy method to analyze the coupled vibration of the thin-walled piezoelectric cylindrical transducers for various polarization states [18,19]. Shuyu Lin utilized the mechanical coupling coefficient method [20,21] to analyze the coupled vibration of the axially polarized piezoelectric cylindrical transducer [22,23] and the elastic cylindrical transducer [24]. However, fewer researchers have studied the radially polarized piezoelectric cylindrical transducer with the mechanical coupling coefficient method.

The object of this paper is the coupled vibration of radially polarized piezoelectric cylindrical transducer, in the hypothesis of axial symmetry modes and neglecting the shear and torsion modes. Generally speaking, numerical simulation methods can analyze the complex coupled vibration of the piezoelectric transducer. However, it is of less physical insight, and more time consuming, compared with the analytical method. In the present paper the radially polarized piezoelectric cylindrical transducer is analyzed with the mechanical coupling coefficient method. The method is an approximate analytical method. A mechanical coupling coefficient is defined as a ratio of radial strain and circumferential strain to axial strain. It divides the piezoelectric equations into the radial and longitudinal vibrational parts. The electric admittances, as functions of resonance frequencies and mechanical coupling coefficients, are derived. The radial and longitudinal vibrational resonance frequency equations are obtained. The effects of aspect ratios on resonance frequencies and mechanical coupling coefficients are studied [25]. In experiment, vibrational distributions on the surfaces of the cylindrical transducer are presented.

## 2. Coupled Vibration Analyses of the Radially Polarized Piezoelectric Cylindrical Transducer

**Figure 1:**
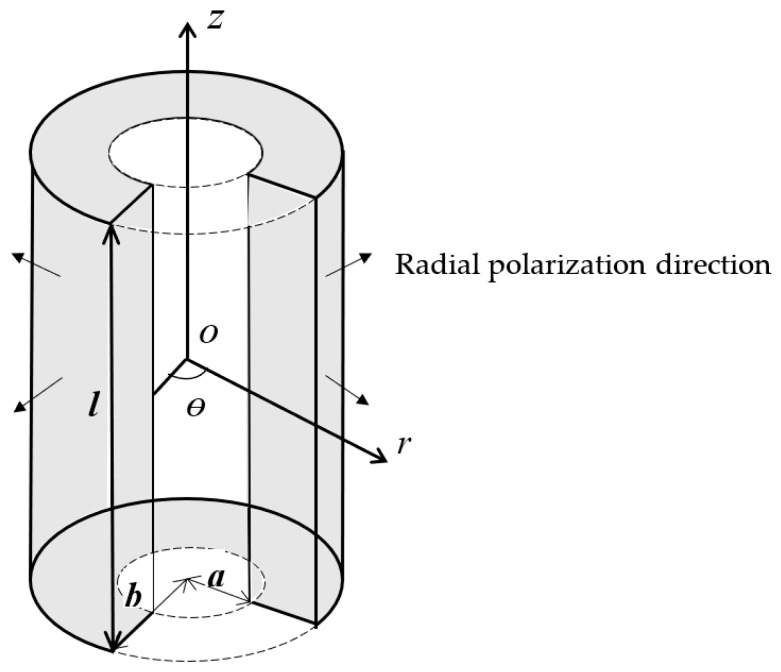
A radially polarized piezoelectric cylindrical transducer.

A piezoelectric cylindrical transducer polarized in the radial direction is shown in Figure 1. In the cylindrical coordinate system, *a*, *b*, and *l* represent the inner radius, the outer radius, and the length. The piezoelectric transducer is considered as mechanically unloaded. The polarization direction is along the plane radial direction and it is denoted by the four arrows. The external exciting electric field is parallel to the polarization direction.

The three-dimensional motion equations for the cylinder in coupled vibration are:

(1a) $$\rho \frac{\partial^{2}\xi_{r}}{\partial t^{2}}=\frac{\partial T_{r}}{\partial r}+\frac{1}{r}\frac{\partial T_{r\theta }}{\partial \theta }+\frac{\partial T_{rz}}{\partial z}+\frac{T_{r}-T_{\theta }}{r}$$

(1b) $$\rho \frac{\partial^{2}\xi_{\theta }}{\partial t^{2}}=\frac{\partial T_{r\theta }}{\partial r}+\frac{1}{r}\frac{\partial T_{\theta }}{\partial \theta }+\frac{\partial T_{\theta z}}{\partial z}+\frac{2T_{r\theta }}{r}$$

(1c) $$\rho \frac{\partial^{2}\xi_{z}}{\partial t^{2}}=\frac{\partial T_{rz}}{\partial r}+\frac{1}{r}\frac{\partial T_{\theta z}}{\partial \theta }+\frac{\partial T_{z}}{\partial z}+\frac{T_{rz}}{r}$$

where *r*, *θ*, *z* are the cylindrical co-ordinates; $\xi_{r}$, $\xi_{\theta }$, $\xi_{z}$ are the three displacement components; and $T_{r}$, $T_{\theta }$, $T_{z}$, $T_{r\theta }$, $T_{rz}$, $T_{\theta z}$ are the stress components in the piezoelectric ceramic cylinder. The relationships between the strain and the displacement are as follows:

(2a) $$S_{r}=\frac{\partial \xi_{r}}{\partial r},\;S_{\theta }=\frac{1}{r}\frac{\partial \xi_{\theta }}{\partial \theta }+\frac{\xi_{r}}{r},\;S_{z}=\frac{\partial \xi_{z}}{\partial z}$$

(2b) $$S_{r\theta }=\frac{1}{r}\frac{\partial \xi_{r}}{\partial \theta }+\frac{\partial \xi_{\theta }}{\partial r}-\frac{\xi_{\theta }}{r},\;S_{\theta z}=\frac{1}{r}\frac{\partial \xi_{z}}{\partial \theta }+\frac{\partial \xi_{\theta }}{\partial z},\;S_{rz}=\frac{\partial \xi_{r}}{\partial z}+\frac{\partial \xi_{z}}{\partial r}$$

Here $S_{r}$, $S_{\theta }$, $S_{z}$, $S_{r\theta }$, $S_{rz}$, $S_{\theta z}$ are the strain components.

The constitutive piezoelectric equations for the radially polarized cylinder are as follows:

(3a) $$T_{\theta }=c_{11}^{E}S_{\theta }+c_{12}^{E}S_{z}+c_{13}^{E}S_{r}-e_{31}E_{r}$$

(3b) $$T_{r}=c_{13}^{E}S_{\theta }+c_{13}^{E}S_{z}+c_{33}^{E}S_{r}-e_{33}E_{r}$$

(3c) $$T_{z}=c_{12}^{E}S_{\theta }+c_{11}^{E}S_{z}+c_{13}^{E}S_{r}-e_{31}E_{r}$$

(3d) $$T_{zr}=c_{44}^{E}S_{zr}$$

(3e) $$D_{r}=e_{31}S_{\theta }+e_{31}S_{z}+e_{33}S_{r}+\varepsilon_{33}^{S}E_{r}$$

(3f) $$D_{z}=e_{15}S_{zr}$$

As the polarization direction is parallel to that of the electric field, the vibration is regarded as a coupled one of longitudinal and radial extensional vibrations, approximately; therefore, shearing and torsion stress and strain can be ignored. In this case, when the edge effect of the electric field is ignored, the following equations are obtained as:

(4a) $$E_{z}=0,\;E_{\theta }=0,\;E_{r}\neq 0,\;D_{z}=0,\;D_{\theta }=0,\;D_{r}\neq 0,$$

(4b) $$T_{rz}=T_{r\theta }=T_{\theta z}=0,\;S_{rz}=S_{r\theta }=S_{\theta z}=0$$

where $E_{z}$, $E_{\theta }$, $E_{r}$ and $D_{z}$, $D_{\theta }$, $D_{r}$ are the electric field and electric displacement components [26].

Therefore, the linear constitutive piezoelectric equations for the radially polarized cylinder are expressed as:

(5a) $$T_{\theta }=c_{11}^{E}S_{\theta }+c_{12}^{E}S_{z}+c_{13}^{E}S_{r}-e_{31}E_{r}$$

(5b) $$T_{r}=c_{13}^{E}S_{\theta }+c_{13}^{E}S_{z}+c_{33}^{E}S_{r}-e_{33}E_{r}$$

(5c) $$T_{z}=c_{12}^{E}S_{\theta }+c_{11}^{E}S_{z}+c_{13}^{E}S_{r}-e_{31}E_{r}$$

(5d) $$D_{r}=e_{31}S_{\theta }+e_{31}S_{z}+e_{33}S_{r}+\varepsilon_{33}^{S}E_{r}$$

where $c_{ij}$, $e_{ij}$ and $\varepsilon_{ij}$ (*i*, *j* = 1, 2, 3) denote elastic, piezoelectric, and dielectric material constants.

The strains are linked to the displacement and radius by gradient relations.

(6) $$S_{r}=\frac{\partial \xi_{r}}{\partial r},\;S_{\theta }=\frac{\xi_{r}}{r},\;S_{z}=\frac{\partial \xi_{z}}{\partial z}$$

Radial and longitudinal motion equations are:

(7a) $$\rho \frac{\partial^{2}\xi_{r}}{\partial t^{2}}=\frac{\partial T_{r}}{\partial r}+\frac{T_{r}-T_{\theta }}{r}$$

(7b) $$\rho \frac{\partial^{2}\xi_{z}}{\partial t^{2}}=\frac{\partial T_{z}}{\partial z}$$

Based on the piezoelectric equations and motion equations, in order to connect the radial vibration with longitudinal vibration, the mechanical coupling coefficient *c* is defined as,

(8) $$c=\frac{c_{13}^{E}S_{z}}{c_{12}^{E}S_{\theta }+c_{13}^{E}S_{r}}$$

The introduction of the mechanical coupling coefficient divides the coupled vibration of the piezoelectric cylindrical transducer into a short ring radial vibration and a long tube longitudinal vibration. In the following analyses, equivalent radial vibration and longitudinal vibration are presented, respectively.

### 2.1. The Equivalent Radial Vibration of the Piezoelectric Cylindrical Transducer in Coupled Vibration

For the radial vibration of the piezoelectric cylindrical transducer, since there are no free moving electrical charges in the piezoelectric ceramic material, the radial electric displacement $D_{r}$ satisfies the electrostatic condition:

(9) $$\frac{1}{r}\frac{\partial }{\partial r}\left(r\cdot D_{r}\right)=0$$

The solution to Equation (9) is:

(10) $$D_{r}=D_{ro}e^{j\omega t}$$

where $D_{ro}=\frac{C^{\prime }}{r}$ is dependent of radius *r*. Then the radial electric field is expressed as:

(11) $$E_{r}=c_{3}S_{\theta }+c_{5}S_{r}+\frac{C^{\prime }}{\varepsilon_{33}^{S}r}$$

where $c_{3}$, $c_{5}$ and $C^{\prime }$ are constants.

Substituting Equations (8) and (11) into Equation (5d), the radial electric displacement can be obtained:

(12) $$D_{r}=(e_{31}+\varepsilon_{33}^{S}c_{3}+e_{31}\frac{c\cdot c_{12}^{E}}{c_{13}^{E}})S_{\theta }+(e_{33}+\varepsilon_{33}^{S}c_{5}+e_{31}c)S_{r}+\frac{C^{\prime }}{r}$$

From Equations (10) and (12), the constants in Equation (11) can be obtained,

(13a) $$c_{3}=\frac{-c_{13}^{E}e_{31}-c\cdot c_{12}^{E}e_{31}}{c_{13}^{E}\varepsilon_{33}^{S}}$$

(13b) $$c_{5}=\frac{-c\cdot e_{31}-e_{33}}{\varepsilon_{33}^{S}}$$

Therefore, the stress expressions of Equation (5a,b) are presented as follows:

(14a) $$T_{\theta }=A_{1}S_{\theta }+A_{4}S_{r}-\frac{e_{31}C^{\prime }}{\varepsilon_{33}^{S}r}$$

(14b) $$T_{r}=A_{2}S_{\theta }+A_{3}S_{r}-\frac{e_{33}C^{\prime }}{\varepsilon_{33}^{S}r}$$

where $A_{1}=c_{11}^{E}+\frac{c\cdot c_{12}^{E}c_{12}^{E}}{c_{13}^{E}}-e_{31}c_{3}$, $A_{2}=c_{13}^{E}+c\cdot c_{12}^{E}-e_{33}c_{3}$, $A_{3}=c_{33}^{E}+c\cdot c_{13}^{E}-e_{33}c_{5}$, $A_{4}=c_{13}^{E}+c\cdot c_{12}^{E}-e_{31}c_{5}$.

Substituting Equation (14a,b) into Equation (7a) yields the motion equation for radial vibration:

(15) $$\frac{\partial^{2}\xi_{r}}{\partial r^{2}}+\frac{m}{r}\frac{\partial \xi_{r}}{\partial r}+(k_{r}^{2}-\frac{v^{2}}{r^{2}})\xi_{r}+\frac{n\cdot C^{\prime }}{r^{2}}=0$$

Here $m=\frac{A_{2}+A_{3}-A_{4}}{A_{3}}$, $n=\frac{e_{31}}{\varepsilon_{33}^{S}A_{3}}$ and $v=\sqrt{\frac{A_{1}}{A_{3}}}$. $k_{r}=\frac{\omega }{V_{r}}$ is the equivalent radial wave number; *ω* is the angular frequency, and $V_{r}=\sqrt{\frac{A_{3}}{\rho }}$ is the equivalent radial sound speed.

By means of the mathematical software of Mathematica 9.0 (Wolfram Research, Champagne, IL, USA), the solution to Equation (15) can be obtained as:

(16) $$\xi_{r}=r^{-\frac{m-1}{2}}(C_{1}\cdot J_{q}(k_{r}r)+C_{2}\cdot Y_{q}(k_{r}r)-C^{\prime }\cdot \frac{n\pi }{2}F_{q}(k_{r}r))$$

where $J_{q}(k_{r}r)$ and $Y_{q}(k_{r}r)$ are Bessel functions of the first kind and the second kind, $q=\sqrt{{\left(\frac{m-1}{2}\right)}^{2}+v^{2}}$. $C_{1}$ and $C_{2}$ are constants determined by the boundary conditions. $F_{q}(k_{r}r)$ is a Lommel function, its expression is:

(17) $$F_{q}(k_{r}r)=(\int r^{\frac{m-1}{2}-1}J_{q}(k_{r}r)dr)\cdot Y_{q}(k_{r}r)-(\int r^{\frac{m-1}{2}-1}Y_{q}(k_{r}r)dr)\cdot J_{q}(k_{r}r)$$

From Equation (16), the radial strain is obtained,

(18) $$\begin{array}{l} \frac{\partial \xi_{r}}{\partial r}=-\frac{m-1}{2}\cdot r^{-\frac{m-1}{2}-1}\left(C_{1}\cdot J_{q}(k_{r}r)+C_{2}\cdot Y_{q}(k_{r}r)-C^{\prime }\cdot \frac{n\pi }{2}F_{q}(k_{r}r)\right)\\ +r^{-\frac{m-1}{2}}(C_{1}\cdot {J_{q}}^{\prime }(k_{r}r)+C_{2}\cdot {Y_{q}}^{\prime }(k_{r}r)-C^{\prime }\cdot \frac{n\pi }{2}{F_{q}}^{\prime }(k_{r}r)) \end{array}$$

The radial stress can be expressed as:

(19) $$T_{r}=A_{2}S_{\theta }+A_{3}S_{r}-\frac{C^{\prime }e_{33}}{\varepsilon_{33}^{S}r}=r^{-\frac{m-1}{2}}[C_{1}\cdot J(r)+C_{2}\cdot Y(r)-C^{\prime }\cdot \frac{n\pi }{2}F(r)]-\frac{C^{\prime }e_{33}}{\varepsilon_{33}^{S}r}$$

where:

$$J(r)=(A_{2}-\frac{m-1}{2}A_{3}+qA_{3})J_{q}(k_{r}r)/r-k_{r}A_{3}J_{q+1}(k_{r}r)$$

$$Y(r)=(A_{2}-\frac{m-1}{2}A_{3}+qA_{3})Y_{q}(k_{r}r)/r-k_{r}A_{3}Y_{q+1}(k_{r}r)$$

$$F(r)=(A_{2}-\frac{m-1}{2}A_{3}+qA_{3})F_{q}(k_{r}r)/r-k_{r}A_{3}F_{q+1}(k_{r}r)$$

The following formulas are utilized in the derivation [27],

$${J_{q}}^{\prime }(k_{r}r)=\frac{q}{r}J_{q}(k_{r}r)-k_{r}J_{q+1}(k_{r}r)$$

$${Y_{q}}^{\prime }(k_{r}r)=\frac{q}{r}Y_{q}(k_{r}r)-k_{r}Y_{q+1}(k_{r}r)$$

$${F_{q}}^{\prime }(k_{r}r)=\frac{q}{r}F_{q}(k_{r}r)-k_{r}F_{q+1}(k_{r}r)$$

$$F_{q+1}(k_{r}r)=(\int r^{\frac{m-1}{2}-1}J_{q}(k_{r}r)dr)\cdot Y_{q+1}(k_{r}r)-(\int r^{\frac{m-1}{2}-1}Y_{q}(k_{r}r)dr)\cdot J_{q+1}(k_{r}r)$$

The unknown constants $C_{1}$ and $C_{2}$ in Equation (16) are determined from the following boundary conditions,

(20a) $$T_{r=a}=0$$

(20b) $$T_{r=b}=0$$

Therefore, $C_{1}$ and $C_{2}$ are obtained,

(21a) $$C_{1}=\frac{C^{\prime }\cdot [a^{\frac{m-1}{2}-1}Y(b)-b^{\frac{m-1}{2}-1}Y(a)]\frac{e_{33}}{\varepsilon_{33}^{S}}+\frac{n\pi }{2}[F(a)Y(b)-F(b)Y(a)]}{J(a)Y(b)-J(b)Y(a)}$$

(21b) $$C_{2}=\frac{C^{\prime }\cdot [a^{\frac{m-1}{2}-1}J(b)-b^{\frac{m-1}{2}-1}J(a)]\frac{e_{33}}{\varepsilon_{33}^{S}}+\frac{n\pi }{2}[F(a)J(b)-F(b)J(a)]}{J(b)Y(a)-J(a)Y(b)}$$

Based on the radial voltage $V_{11}=\int_{a}^{b}E_{r}dr$ and the radial current $I_{11}=j\omega \oint D_{r}dS$, the electric admittance of the radial vibration is obtained:

(22) $$Y_{11}=\frac{I_{11}}{V_{11}}=\frac{j\omega \oint D_{r}dS}{\int_{a}^{b}E_{r}dr}=\frac{j\omega 2\pi l}{P\cdot J_{ba}+Q\cdot Y_{ba}-\frac{n\pi }{2}F_{ba}+\frac{Inb-Ina}{\varepsilon_{33}^{S}}}$$

where:

$$P=\frac{\left(a^{\frac{m-1}{2}-1}\cdot Y(b)-b^{\frac{m-1}{2}-1}\cdot Y(a))\frac{e_{33}}{\varepsilon_{33}^{S}}+\frac{n\pi }{2}(F(a)Y(b)-F(b)Y(a)\right)}{J(a)Y(b)-J(b)Y(a)}$$

$$Q=\frac{\left(a^{\frac{m-1}{2}-1}\cdot J(b)-b^{\frac{m-1}{2}-1}\cdot J(a))\frac{e_{33}}{\varepsilon_{33}^{S}}+\frac{n\pi }{2}(F(a)J(b)-F(b)J(a)\right)}{J(b)Y(a)-J(a)Y(b)}$$

$$J_{ba}=(c_{3}-\frac{m-1}{2}\cdot c_{5}+qc_{5})\int_{a}^{b}r^{-\frac{m-1}{2}-1}J_{q}(k_{r}r)dr-k_{r}\int_{a}^{b}r^{-\frac{m-1}{2}}J_{q+1}(k_{r}r)dr$$

$$Y_{ba}=(c_{3}-\frac{m-1}{2}\cdot c_{5}+qc_{5})\int_{a}^{b}r^{-\frac{m-1}{2}-1}Y_{q}(k_{r}r)dr-k_{r}\int_{a}^{b}r^{-\frac{m-1}{2}}Y_{q+1}(k_{r}r)dr$$

$$F_{ba}=(c_{3}-\frac{m-1}{2}\cdot c_{5}+qc_{5})\int_{a}^{b}r^{-\frac{m-1}{2}-1}F_{q}(k_{r}r)dr-k_{r}\int_{a}^{b}r^{-\frac{m-1}{2}}F_{q+1}(k_{r}r)dr$$

### 2.2. The Equivalent Longitudinal Vibration of the Piezoelectric Cylindrical Transducer in Coupled Vibration

For the longitudinal vibration of the piezoelectric cylindrical transducer, based on Equations (8), (11), (5c) and (7b), the longitudinal displacement is obtained,

(23) $$\xi_{z}=A_{z}\sin(k_{z}z)+B_{z}\cos(k_{z}z)$$

From Equation (23), the longitudinal strain is expressed as:

(24) $$\frac{\partial \xi_{z}}{\partial z}=A_{z}k_{z}\cos(k_{z}z)-B_{z}k_{z}\sin(k_{z}z)$$

The longitudinal stress is expressed as:

(25) $$T_{z}=(\frac{c_{13}^{E}}{c}+c_{11}^{E})S_{z}-e_{31}E_{r}=(\frac{c_{13}^{E}}{c}+c_{11}^{E})[A_{z}k_{z}\cos(k_{z}z)-B_{z}k_{z}\sin(k_{z}z)]-e_{31}E_{r}$$

where the unknown constants $A_{z}$ and $B_{z}$ are determined from the following boundary conditions,

(26a) $$T_{z=0}=0$$

(26b) $$T_{z=l}=0$$

The constants are obtained:

(27a) $$A_{z}=\frac{c\cdot e_{31}E_{r}}{(c_{13}^{E}+c\cdot c_{11}^{E})k_{z}}$$

(27b) $$B_{z}=\frac{c\cdot e_{31}E_{r}}{c_{13}^{E}+c\cdot c_{11}^{E}}\frac{\left(\cos(k_{z}l)-1\right)}{k_{z}\sin(k_{z}l)}$$

For the longitudinal vibration of the cylinder, under quasi-static condition $S_{r}=S_{\theta }$, and the mechanical coupling coefficient is expressed as $c=\frac{c_{13}^{E}S_{z}}{(c_{12}^{E}+c_{13}^{E})S_{r}}$.

The electric displacement is:

(28) $$\begin{array}{l} D_{r}=(e_{31}+e_{33})S_{r}+e_{31}S_{z}+\varepsilon_{33}^{S}E_{r}=\left[\left(e_{31}+e_{33}\right)\frac{c_{13}^{E}}{c(c_{12}^{E}+c_{13}^{E})}+e_{31}\right]S_{z}+\varepsilon_{33}^{S}E_{r}\\ =\left[\left(e_{31}+e_{33}\right)\frac{c_{13}^{E}}{c(c_{12}^{E}+c_{13}^{E})}+e_{31}\right]\left[\frac{c\cdot e_{31}E_{r}}{c_{13}^{E}+c\cdot c_{11}^{E}}\left(\cos\left(k_{z}z\right)-\frac{\cos\left(k_{z}l\right)-1}{\sin(k_{z}l)}\sin\left(k_{z}z\right)\right)\right]+\varepsilon_{33}^{S}E_{r} \end{array}$$

Here, the radial electric field $E_{r}$ is considered as a constant.

Based on the longitudinal voltage $V_{13}=\int_{a}^{b}E_{r}dr$ and the current $I_{13}=j\omega \oint D_{r}dS$, the electric admittance of longitudinal vibration is:

(29) $$Y_{13}=\frac{I_{13}}{V_{13}}=\frac{j\omega \oint D_{r}dS}{\int_{a}^{b}E_{r}dr}=\frac{j\omega 2\pi l\cdot \left(\frac{a+b}{2}\right)\left[(\left(e_{31}+e_{33}\right)\frac{c_{13}^{E}}{c(c_{12}^{E}+c_{13}^{E})}+e_{31})\frac{c\cdot e_{31}}{c_{13}^{E}+c\cdot c_{11}^{E}}\frac{\tan\left(k_{z}l/2\right)}{k_{z}l}+\varepsilon_{33}^{S}\right]}{b-a}$$

It is found that Equations (22) and (29) are closely related with material parameters, geometrical dimensions, the mechanical coupling coefficient, and the frequency. When the material parameters and the geometrical dimension are given, $Y_{11}$ and $Y_{13}$ are the functions of the mechanical coupling coefficient and the frequency. When $Y_{11}$ and $Y_{13}$ both reach the maximum, resonance frequency equations are obtained. For the resonance frequency equations, there are two groups of solutions. One group is the radial resonance frequency and the corresponding mechanical coupling coefficient. The other group is the longitudinal resonance frequency and the corresponding mechanical coupling coefficient.

## 3. The Dependence of Resonance Frequency and Mechanical Coupling Coefficient on Aspect Ratio

To analyze the dependence of the resonance frequency and the mechanical coupling coefficient on the aspect ratio, Equations (22) and (29) are solved. Since they are complex transcendental functions, it is difficult to obtain the analytical solutions and Mathematica 9.0 (Wolfram Research, Champagne, IL, USA) software is used to calculate the equations.

For the piezoelectric cylindrical transducer, the inner radius *a* = 0.016 m and outer radius *b* = 0.02 m are fixed, and the length *l* is changed. The piezoelectric material used is an equivalent of PZT-4 (Kunshan Risun Electronic Co., Ltd. Kunshan, China) manufactured in China. The relevant standard parameters of PZT-4 are listed in Table 1 [28].

**Figure 2:**
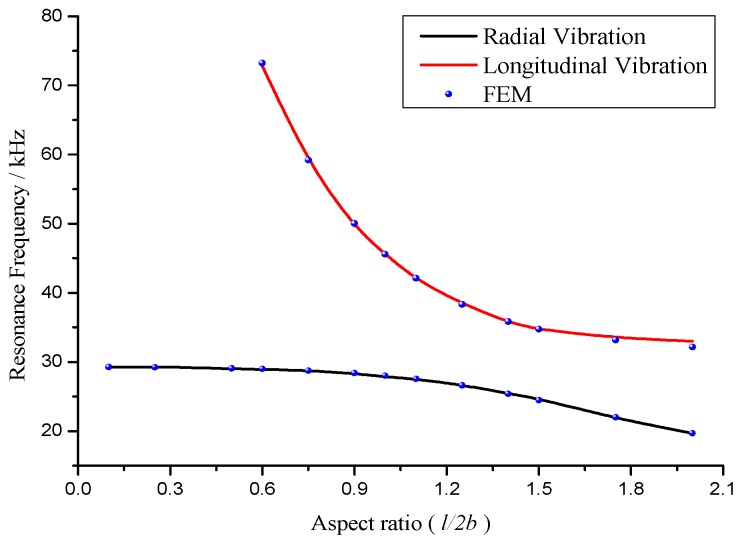
The relationships between resonance frequency and aspect ratio.

**Figure 3:**
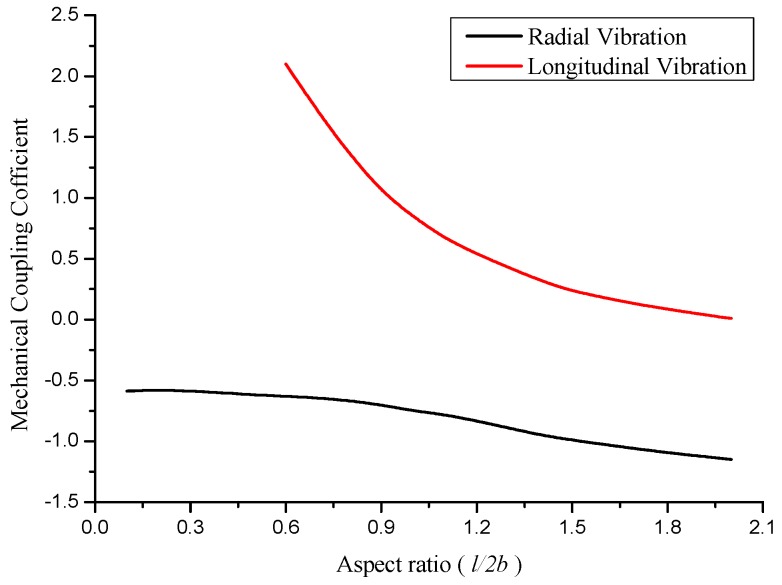
The relationships between mechanical coupling coefficient and aspect ratio.

When the aspect ratio *l*/*2b* is increasing from 0.1, the resonance frequencies and the corresponding mechanical coupling coefficients are calculated and illustrated in Figure 2 and Figure 3.

Figure 2 demonstrates the dependence of the radial and longitudinal resonance frequencies on aspect ratio. It is seen that when the aspect ratio increases, the radial and longitudinal resonance frequencies decrease. The influence of longitudinal dimension on longitudinal resonance frequency is stronger than that on the radial resonance frequency. At the aspect ratio of 0.9 < *l*/*2b* < 1.5, the radial and longitudinal resonance frequencies are close. It is considered that there is a strong coupling between the radial and longitudinal partial systems.

In addition, the finite element method (FEM) is used to compute the radial and longitudinal resonance frequencies on different aspect ratios. The simulated resonance frequencies are illustrated in Figure 2. It is found that the theoretical results are in good agreement with the simulated results.

Figure 3 demonstrates the effect of aspect ratio on the corresponding radial and longitudinal mechanical coupling coefficients. From the resonance frequency equations, two kinds of mechanical coupling coefficients are found. The negative mechanical coupling coefficient corresponds to the radial vibration, and the positive to the longitudinal vibration. When the aspect ratio increases, the values of mechanical coupling coefficients decrease. At the aspect ratio of 0.9 < *l*/*2b* < 1.5, the mechanical coupling coefficients are at the vicinity of ±1. The coupling of radial and longitudinal vibration is strong. At the aspect ratio of *l*/*2b* < 0.9 and *l*/*2b* > 1.5, the coupled vibration is considered weak.

It is concluded that when the radial and longitudinal resonance frequencies are far away from each other, the coupled vibration is weak. When the radial and longitudinal resonance frequencies are close, the coupled vibration is strong and the mechanical coupling coefficient is at the vicinity of ±1.

In addition, COMSOL Multiphysics 5.2 (COMSOL Inc. Stockholm, Sweden) is used to simulate the different coupled vibrational modes on different aspect ratios. The piezoelectric devices in the structural mechanics and the eigenfrequency type of analysis are selected, respectively. A radially polarized piezoelectric cylinder is structured and the material of the model is the lead zirconate titanate (PZT-4). The mesh density is set as 20. The elastic boundary condition is set as ‘free’. The low voltage is applied on the inside surface and outside surface is grounded.

**Figure 4:**
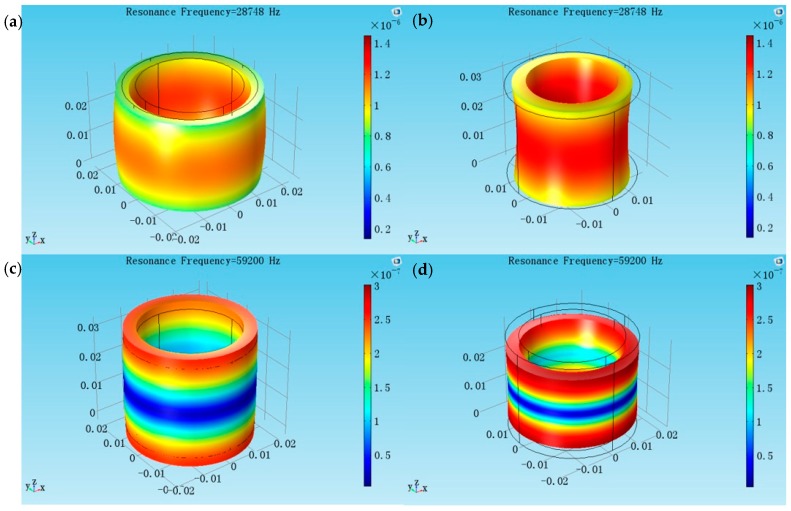
The coupled vibration modes of the cylindrical transducer at aspect ratio of *l*/*2b* = 0.75. (**a**,**b**) The radial extensional vibration mode of the coupled vibration; (**c**,**d**) The longitudinal extensional vibration mode of the coupled vibration.

**Figure 5:**
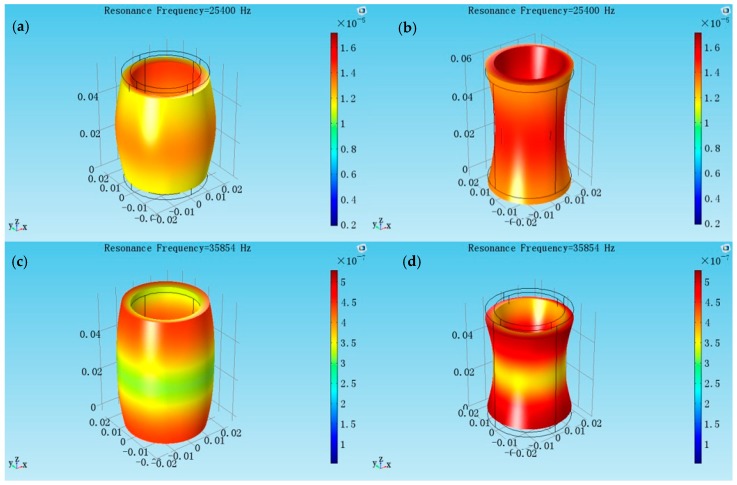
The coupled vibration modes of the cylindrical transducer at aspect ratio of *l*/*2b* = 1.4. (**a**,**b**) The radial extensional vibration mode of the coupled vibration; (**c**,**d**) The longitudinal extensional vibration mode of the coupled vibration.

Two radially polarized piezoelectric cylindrical transducer models are created separately, the inner and outer radii are the same (*a* = 0.016 m, *b* = 0.02 m), but the lengths are different (*l* = 0.03 m and *l* = 0.056 m). Figure 4 shows at the aspect ratio *l*/*2b* = 0.75, the radial resonance frequency is 28.748 kHz, and the longitudinal resonance frequency is 59.200 kHz. From the vibrational modes, the coupling between the radial and longitudinal vibration is weak. Figure 5 shows at the aspect ratio *l*/*2b* = 1.4, the radial resonance frequency is 25.400 kHz and the longitudinal resonance frequency is 35.854 kHz. From the vibrational modes, the radial vibration and longitudinal vibrations affect each other strongly. It is seen that the aspect ratio affects the coupled degree of the cylindrical transducer.

## 4. Experiments

To verify the theoretical analyses of coupled vibration, a radially polarized piezoelectric cylindrical transducer made of PZT-4, with the inner radius *a* = 0.016 m, the outer radius *b* = 0.02 m, and the length *l* = 0.03 m was manufactured. The resonance frequencies of the cylindrical transducer are measured by the Polytec-400 scanning vibrometer (Polytec, Waldbronn, Germany). The measured results are listed in Table 2.

In Table 2, the experiment method, the mechanical coupling coefficient method, and the finite element method are used, and the radial and longitudinal resonance frequencies are presented. The theoretical results are calculated by Mathematica 9.0 (Wolfram Research, Champagne, IL, USA) according to Equations (22) and (29). The results from the finite element method are calculated with COMSOL Multiphysics 5.2 (COMSOL Inc., Stockholm, Sweden), as the previous section presented.

It is seen that the mechanical coupling coefficient method results are in good agreement with the experimental results and the finite element method results.

**Figure 6:**
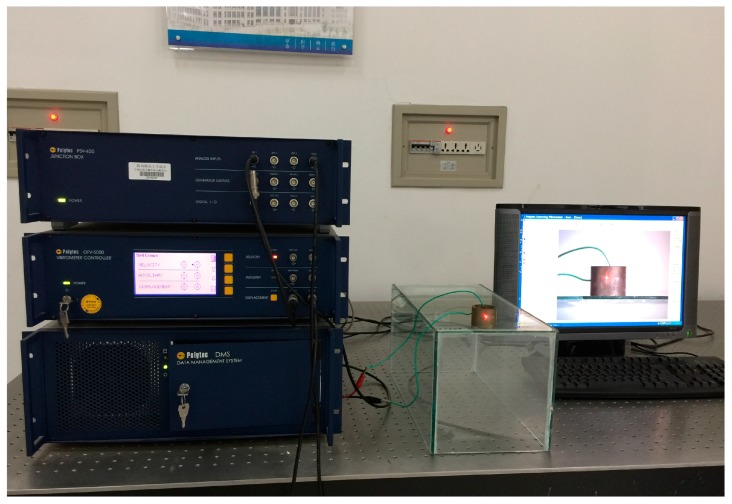
Experimental setup for the measurement of the radially polarized piezoelectric cylindrical transducer.

**Figure 7:**
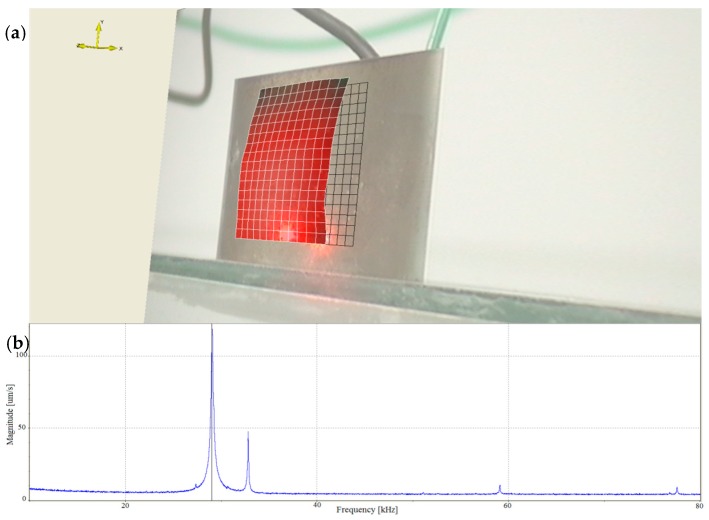
(**a**) The radial vibrational distribution on the side surface of the cylindrical transducer at the radial resonance frequency; (**b**) The frequency-radial vibrational velocity curve.

**Figure 8:**
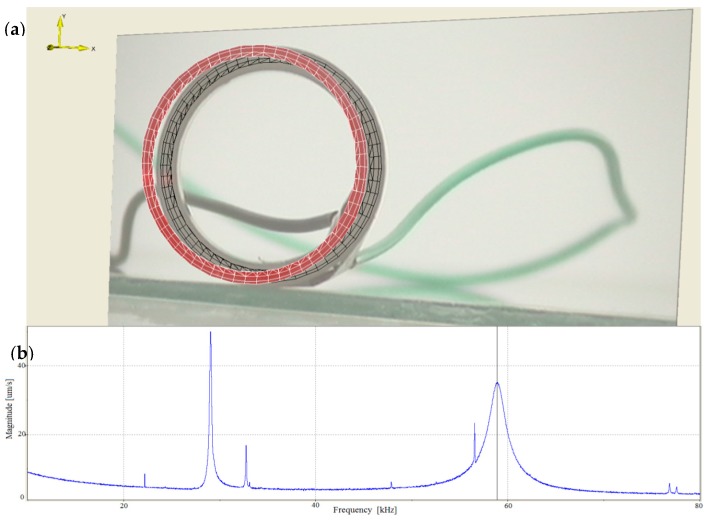
(**a**) The longitudinal vibrational distribution on the end surface of the cylindrical transducer at the longitudinal resonance frequency; (**b**) The frequency-longitudinal vibrational velocity curve.

In addition, to present the surface vibrational distributions of the piezoelectric cylindrical transducer in coupled vibrations, Polytec scanning vibrometer (Polytec, Waldbronn, Germany) is used. In the measurement, a frequency-sweeping electric signal produced by Polytec OFV-5000 vibrometer controller is applied to the inside and outside surfaces of the piezoelectric cylinder. By means of the piezoelectric effect, mechanical vibration is produced and it excites the cylinder to vibrate. At the output end of the cylindrical transducer, Polytec PSV-400 laser scanning head automatically measures the vibration distribution on the surfaces. The experimental setup of the piezoelectric transducer is illustrated in Figure 6. The vibrational displacement distributions and frequency responses of the cylindrical transducer are recorded in Figure 7 and Figure 8, respectively. Figure 7a presents the radial vibrational distribution on the side surface of the transducer at the first resonance frequency. Figure 7b is the frequency-radial vibrational velocity curve. In the frequency response pattern, the frequency corresponding to the velocity peak is the radial resonance frequencies of the cylindrical transducer. Figure 8a illustrates the longitudinal vibrational distribution on the end surface of the cylindrical transducer at the longitudinal resonance frequency. Figure 8b is the frequency-longitudinal vibrational velocity curve. In the frequency response pattern, the frequency corresponding to the velocity peak is the longitudinal resonance frequency of the cylindrical transducer.

## 5. Conclusions

Coupled vibration of the radially polarized piezoelectric cylindrical transducer is analyzed based on the mechanical coupling coefficient method. The introduction of mechanical coupling coefficient divides the coupled vibration into the equivalent radial and longitudinal vibrations. By the theoretical derivation, the electric admittances as functions of mechanical coupling coefficients and resonance frequencies are obtained. The radial and longitudinal resonance frequency equations are acquired.

The dependence of resonance frequency and mechanical coupling coefficient on the aspect ratio of the transducer is analyzed. At the aspect ratio of 0.9 < *l*/*2b* < 1.5, the radial and longitudinal resonance frequencies are close, and the mechanical coupling coefficients are at the vicinity of ±1. It is considered that the coupling of the radial and longitudinal vibrations is strong. At the aspect ratio of *l*/*2b* < 0.9 and *l*/*2b* > 1.5, the radial and longitudinal resonance frequencies are far away each other, and the coupling of the two parts is weak.

In practical applications, the cylindrical piezoelectric transducer is neither a very thin ring nor a very long, slender cylinder. Therefore, its coupled vibration is significant and cannot be ignored. The analysis in this paper can be effectively used to analyze the coupled vibration. It is an approximately analytical method and can be used to other cases of coupled vibrations of piezoelectric devices which are nowadays widely used in ultrasonic emitters and sensors, such as underwater sound hydrophones, ultrasonic transducers, piezoelectric transformers, and other applications.

## Figures and Tables

**Table 1:** Standard parameters of PZT-4.

Parameters	Value
$\rho \left(kg/m^{3}\right)$	7500
$c_{11}^{E}\left(N/m^{2}\right)$	$13.9\times 10^{10}$
$c_{12}^{E}\left(N/m^{2}\right)$	$7.78\times 10^{10}$
$c_{13}^{E}\left(N/m^{2}\right)$	$7.43\times 10^{10}$
$c_{33}^{E}\left(N/m^{2}\right)$	$13.9\times 10^{10}$
$e_{31}\left(N/\left(m/V\right)\right)$	−5.2
$e_{33}\left(N/\left(m/V\right)\right)$	15.1
$\varepsilon_{33}^{S}\left(C/m\right)$	$5.62\times 10^{-9}$

**Table 2:** Comparison of resonance frequencies from different methods.

	Resonance Frequency (kHz)	
	Radial Vibration	Longitudinal Vibration
Experiment measurement	29.063	58.891
Mechanical coupling coefficient method	28.954	58.750
Finite element method	28.748	59.200

## Nomenclature

$\xi_{i}\left(m\right)$	Displacement component
$T_{i}\left(Pa\right)$	Stress component
$S_{i}$	Strain component
$E_{i}\left(V/m\right)$	Electric field component
$D_{i}\left(C/m^{2}\right)$	Electric displacement component
$c_{ij}\left(N/m^{2}\right)$	Elastic material constant
$e_{ij}\left(N/\left(m/V\right)\right)$	Piezoelectric material constant
$\varepsilon_{ij}\left(C/m\right)$	Dielectric material constant
$\rho \left(kg/m^{3}\right)$	Density
c	Mechanical coupling coefficient
$k_{r}$	Equivalent radial wave number
$V_{r}$	Equivalent radial sound speed
$J_{q}(k_{r}r)$	Bessel functions of the first kind
$Y_{q}(k_{r}r)$	Bessel functions of the second kind
$F_{q}(k_{r}r)$	Lommel function
